# Pregnancy outcomes in patients with primary antiphospholipid syndrome: A systematic review and meta-analysis

**DOI:** 10.1097/MD.0000000000015733

**Published:** 2019-05-17

**Authors:** Liping Liu, Dan Sun

**Affiliations:** Department of Obstetrics and Gynecology, Hanchuan People's Hospital, Hanchuan, Hubei, China.

**Keywords:** antiphospholipid syndrome, fetal loss, pregnancy, pregnancy-induced hypertension, pulmonary embolism, thrombosis

## Abstract

**Background:**

Antiphospholipid syndrome (APS) is a rare heterogenous autoimmune disorder with severe life-threatening complications shown during pregnancy. In this analysis, we aimed to systematically compare the pregnancy outcomes (both maternal and fetal) in patients with APS.

**Methods:**

Web of Science, Google Scholar, Medicus, Cochrane Central, Embase, and Medline were searched for relevant English publications. The main inclusion criteria were based on studies that compared pregnancy outcomes in patients with APS vs a control group. Statistical analysis was carried out by the RevMan software version 5.3. This analysis involved dichotomous data, and risk ratios (RR) with 95% confidence intervals (CIs) were used to represent the analysis.

**Results:**

Eight studies consisting of a total number of 212,954 participants were included. Seven hundred seventy participants were pregnant women with APS and 212,184 participants were assigned to the control group. Pregnancy-induced hypertension was significantly higher in women with APS (RR: 1.81, 95% CI: 1.33 – 2.45; *P* = .0002). The risks of fetal loss (RR: 1.33, 95% CI: 1.00–1.76; *P* = .05), abortion (RR: 2.42, 95% CI: 1.46–4.01; *P* = .0006), thrombosis (RR: 2.83, 95% CI: 1.47–5.44; *P* = .002), and preterm delivery (RR: 1.89, 95% CI: 1.52–2.35; *P* = .00001) were also significantly higher in women with APS. However, placental abruption (RR: 1.35, 95% CI: 0.78–2.34; *P* = .29) and pulmonary embolism were not significantly different (RR: 1.47, 95% CI: 0.11–19.20; *P* = .77). The risk of neonatal mortality (RR: 3.95, 95% CI: 1.98–7.86; *P* = .0001), infants small for gestational age (RR: 1.38, 95% CI: 1.04–1.82; *P* = .02), premature infants (RR: 1.86, 95% CI: 1.52–2.28; *P* = .0001), and infants who were admitted to neonatal intensive care unit (RR: 3.35, 95% CI: 2.29–4.89; *P* = .00001) were also significantly higher in women with APS.

**Conclusion:**

This analysis showed APS to be associated with significantly worse pregnancy outcomes when compared to the control group. A significantly higher risk of maternal and fetal complications was observed in this category of patients. Therefore, intense care should be given to pregnant women with APS to monitor unwanted outcomes and allow a successful pregnancy.

## 1 Introduction

Antiphospholipid syndrome (APS) is a rare heterogenous autoimmune disorder which is associated with severe life-threatening complications during pregnancy.^[1]^ This disease affects a minority of women, and is associated with maternal and fetal complications such as miscarriage, eclampsia, fetal intrauterine growth retardation, preterm delivery, and neonatal mortality.^[2]^

During pregnancy, the concentration of coagulation factors increases. However, in pregnant women with APS, this hypercoagulable state including an elevated level of coagulated factors in blood, an increased activated protein C resistance, increased concentration of plasminogen activator inhibitors, and decreased protein S levels might lead to life-threatening complications.

Even though a successful pregnancy is possible in women with APS without treatment, high risks of complications are still possible. Literature reviews have well explained the association of APS with worse pregnancy outcomes. However, an evidence-based analysis has seldom been carried out.

A recent meta-analysis has focused on the impact of systemic lupus erythematosus (SLE) on pregnancy outcomes.^[3]^ The authors clearly demonstrated pregnant women with SLE to have worse maternal and fetal outcomes compared to women who did not have SLE. Another meta-analysis has even compared pregnant women with SLE vs those with APS.^[4]^ However, previous studies did not systematically focus specifically on the impact of APS on pregnancy outcomes.

In this analysis, we aimed to systematically compare the pregnancy outcomes (both maternal and fetal) in patients with primary APS vs a control group.

## 2 Materials and methods

### 2.1 Search databases and search strategies

Web of Science, Google Scholar, Cochrane Central, Medicus, Directory of open access journals, Embase, and PubMed Central/Medline were searched for relevant English publications using the following search terms: “antiphospholipid syndrome and pregnancy,” “antiphospholipid syndrome and maternal outcomes,” “antiphospholipid syndrome and fetal outcomes,” “antiphospholipid syndrome and women,” “APS and pregnancy,” “APS and maternal outcomes,” and “APS and fetal outcomes.”

### 2.2 Inclusion criteria

Inclusion criteria were:

- Studies that compared pregnancy outcomes in patients with APS vs a control group
- English publications

### 2.3 Exclusion criteria

Exclusion criteria were:

- Studies based on pregnancy outcomes in patients with APS without a control group
- Meta-analyses, case studies, literature reviews
- Non-English publications
- Duplicated studies

### 2.4 Data extraction and quality assessment

All relevant data were independently collected by the 2 authors (LLP and SD) and then checked for any error or any missing data.

Any disagreement about including or excluding certain data was carefully discussed with the corresponding author (SD), who was responsible to take a final decision.

Quality assessment of the observational retrospective and prospective studies was carried out by the Newcastle–Ottawa scale (NOS) where scores were given in terms of stars.^[5]^ A maximum number of 9 stars were given indicating a low risk of bias.

### 2.5 Statistical analysis

Statistical analysis was carried out by the RevMan software version 5.3.

This analysis involved dichotomous data, and risk ratios (RRs) with 95% confidence intervals (CIs) were used to represent the analysis.

Heterogeneity was present in this analysis and it was assessed by the Q statistic and the *I*^2^ statistic tests.

A result was considered to be statistically significant if the *P* value was ≤.05.

Heterogeneity based on the *I*^2^ value was represented in percentage. The lower this value, the lower the heterogeneity.

The application of statistical model was based on the *I*^2^ heterogeneity value. A fixed effect model was used if *I*^2^ was ≤50%, or else, a random effect model was used.

Sensitivity analysis was carried out by an exclusion method, whereas publication bias was observed through funnel plots.

### 2.6 Ethical compliances

No ethical or board review approval was required for this analysis.

## 3 Results

### 3.1 Search outcomes

A total of 1320 publications were searched. About 1265 publications were eliminated during an initial assessment. Fifty-five full text publications were assessed. The PRISMA study guideline was followed.^[6]^

Further elimination was carried out:

- Systematic reviews (1)
- Literature reviews (5)
- Compared treatment strategy (6)
- Case studies (5)
- No control group (4)
- Based on non-pregnancy (5)
- Chinese article (1)
- Duplicates (20)

**Figure 1:**
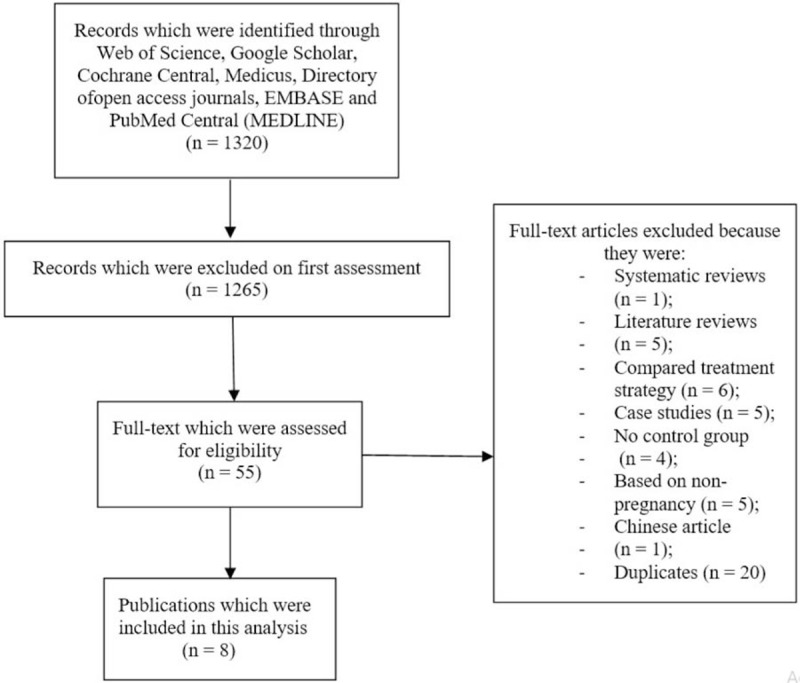
Flow diagram showing the study selection.

Only 8 articles^[7–14]^ were finally included in this meta-analysis, as shown in Figure 1.

### 3.2 General and baseline properties of the studies

The 8 studies consisted of a total number of 212,954 participants. About 770 participants were pregnant women with APS (experimental group) and 212,184 participants were pregnant women who were assigned to the control group, as shown in Table 1. Participants were enrolled between years 1970 and 2015.

**Table 1:**
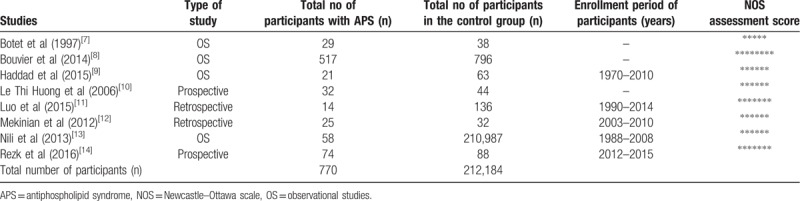
General properties of the studies.

Baseline properties are listed in Table 2. Study Botet et al^[7]^ did not report any baseline feature.

**Table 2:**
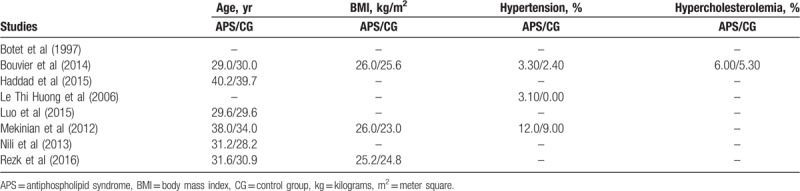
Baseline features of the studies.

The mean age of the pregnant women was 28.2 to 40.2 years. Body mass index varied between 23.0 and 26.0 kg/m^2^. The percentage of pregnant women with hypertension was minimal (0–12.0%) only.

### 3.3 Outcomes which were reported

The maternal and fetal outcomes (Table 3) which were assessed included:

1. Maternal outcomes:
  - Pregnancy-induced hypertension
  - Fetal loss
  - Placental abruption
  - Abortion
  - Thrombosis
  - Preterm delivery
  - Pulmonary embolism
2. Fetal outcomes:
  - Neonatal mortality
  - Infant small for gestational age
  - Premature infants
  - Admission to neonatal intensive care unit (ICU)

**Table 3:**
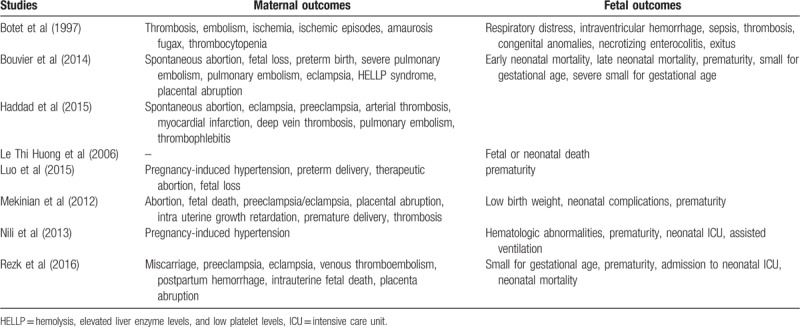
Outcomes which were reported.

### 3.4 Main results of this analysis

**Figure 2:**
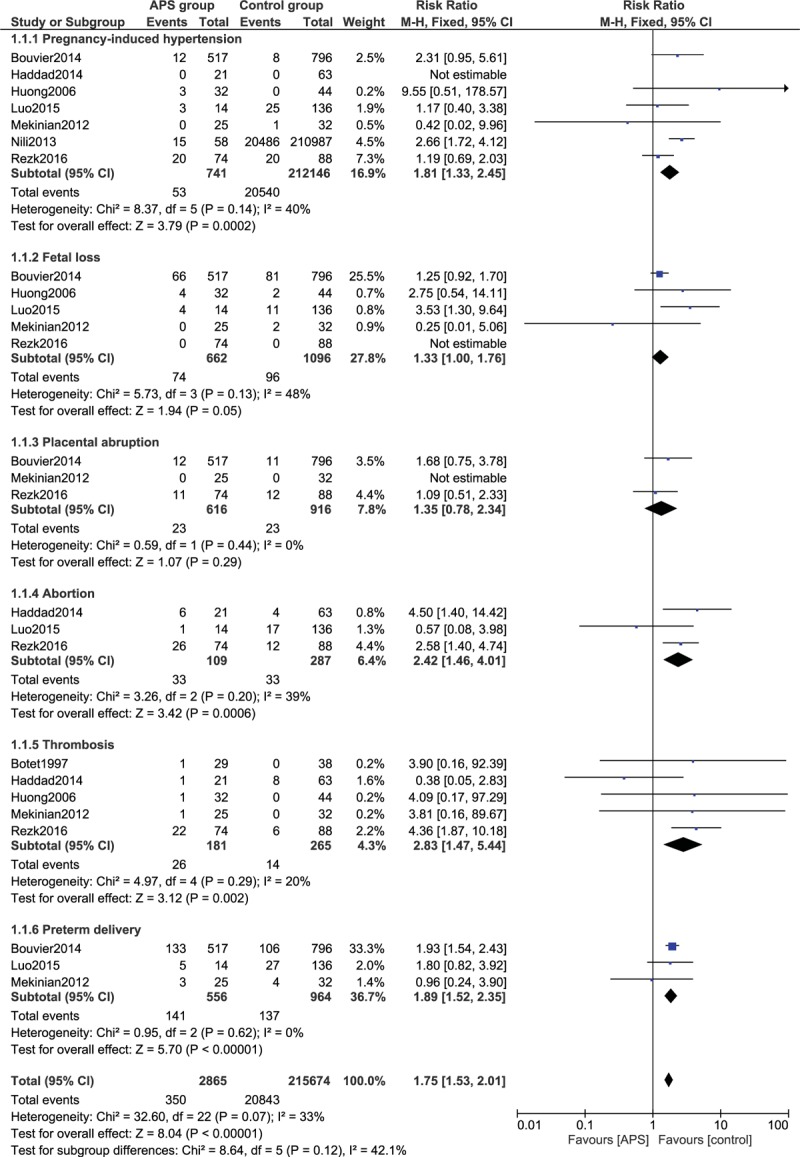
Adverse maternal outcomes observed in pregnant women with antiphospholipid syndrome (Part I). APS = antiphospholipid syndrome, CI = confidence interval.

First of all the maternal outcomes were assessed. Results of this current analysis showed that pregnancy-induced hypertension (eclampsia/preeclampsia) was significantly higher in women with APS (RR: 1.81, 95% CI: 1.33–2.45; *P* = .0002), as shown in Figure 2. The risks of fetal loss (RR: 1.33, 95% CI: 1.00–1.76; *P* = .05), abortion (RR: 2.42, 95% CI: 1.46–4.01; *P* = .0006), thrombosis (RR: 2.83, 95% CI: 1.47–5.44; *P* = .002), and preterm delivery (RR: 1.89, 95% CI: 1.52–2.35; *P* = .00001) were also significantly higher in women with APS (Fig. 2). However, placental abruption (RR: 1.35, 95% CI: 0.78–2.34; *P* = .29) was not significantly different.

**Figure 3:**
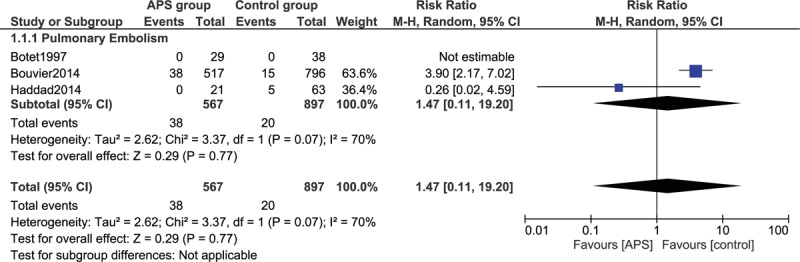
Adverse maternal outcomes observed in pregnant women with antiphospholipid syndrome (Part II). APS = antiphospholipid syndrome, CI = confidence interval.

Our analysis also showed that the risk for pulmonary embolism was not significantly different (RR: 1.47, 95% CI: 0.11–19.20; *P* = .77) in pregnant women with APS vs the control group, as shown in Figure 3.

**Figure 4:**
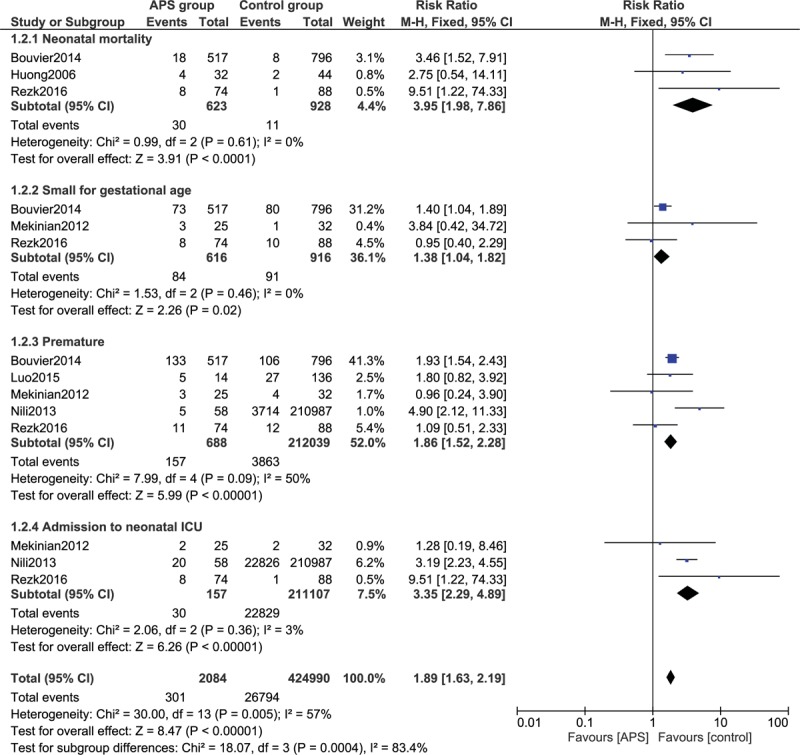
Adverse fetal outcomes observed in pregnant women with antiphospholipid syndrome (Part III). APS = antiphospholipid syndrome, CI = confidence interval, ICU = intensive care unit.

Fetal outcomes were also assessed in this analysis. The current results showed that the risk of neonatal mortality (RR: 3.95, 95% CI: 1.98–7.86; *P* = .0001), infants small for gestational age (RR: 1.38, 95% CI: 1.04–1.82; *P* = .02), premature infants (RR: 1.86, 95% CI: 1.52–2.28; *P* = .0001), and infants who were admitted to neonatal ICU (RR: 3.35, 95% CI: 2.29 – 4.89; *P* = .00001) were significantly higher in women with APS as compared to the control group, as shown in Figure 4.

**Figure 5:**
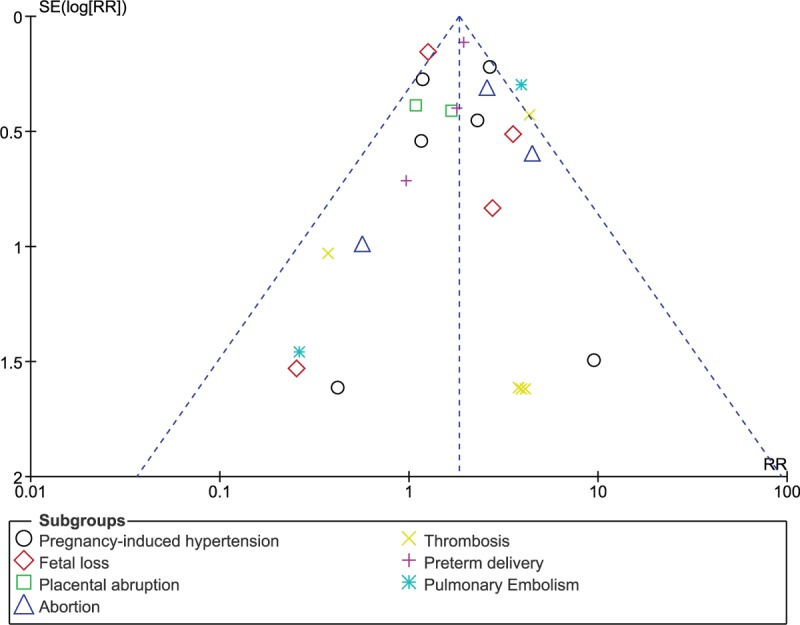
Funnel plot showing publication bias. RR = risk ratio.

**Figure 6:**
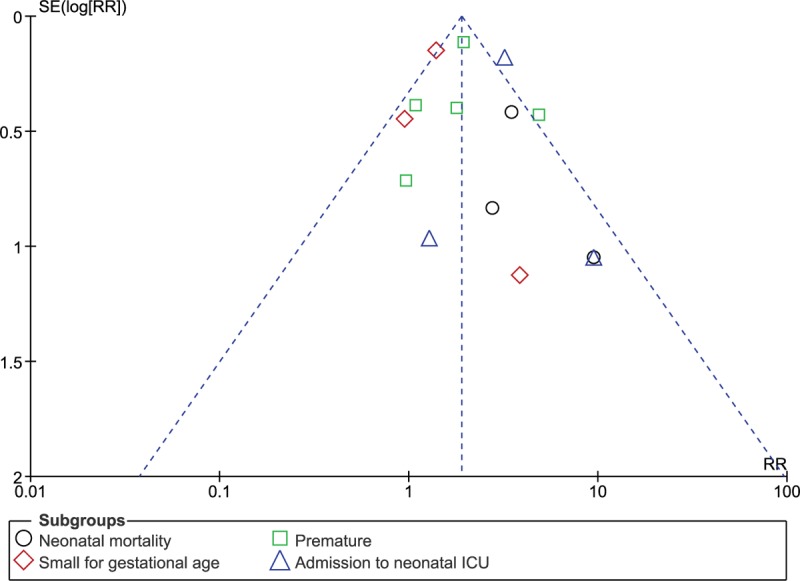
Funnel plot showing publication bias. ICU = intensive care unit, RR = risk ratio.

Detailed results are listed in Table 4. Sensitivity analysis showed consistency throughout. Low evidence of publication bias was observed through the funnel plots, as shown in Figures 5 and 6.

**Table 4:**
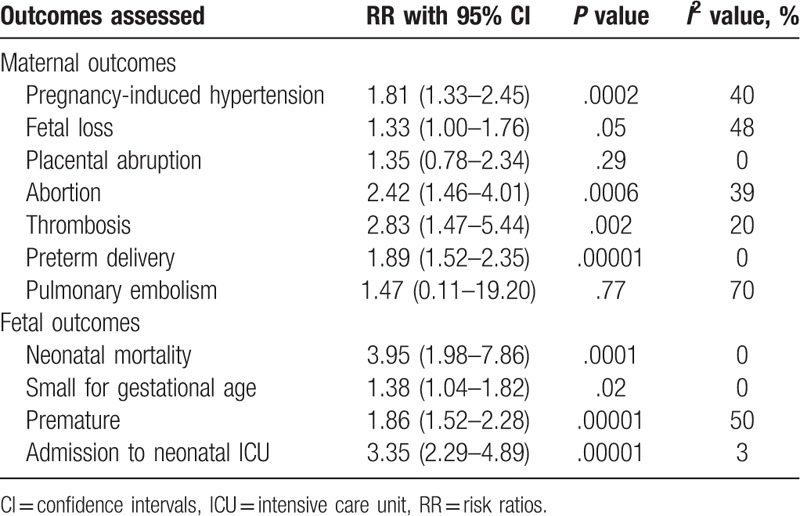
Results of this analysis.

## 4 Discussion

Our study aimed to systematically show with evidence, the impact of APS on pregnancy outcomes. The current results showed APS to be associated with significantly higher risks of pregnancy-induced hypertension, fetal loss, abortion, thrombosis, and preterm delivery. The risk of neonatal mortality, risk of having an infant which is small for gestational age, premature infants, and infants with severe complications who were admitted to neonatal ICU were significantly higher with APS.

This current result is similar to another meta-analysis which assessed the impact of SLE on pregnancy outcomes.^[3]^ The authors showed the number of cesarean operation to be significantly higher in pregnant women with SLE; however, this endpoint was not assessed in our current analysis due to a minimal number of studies reporting this endpoint. Congenital defects were also assessed in these women with SLE. Or similar to their analysis, this current analysis showed APS to be associated with higher risk of infants who were small for gestational age, and who were born prematurely.

Another study involving 15 cases of APS during pregnancy showed 50% of catastrophic APS to appear during pregnancy.^[15]^ Hemolysis, elevated liver enzyme, and low platelets (HELLP syndrome) were seen in 53% of the participants. This study even showed a high rate of maternal and fetal mortality in those pregnant women with APS.

The PREGNANTS study has shown specific antibodies to be associated with obstetric complications.^[16]^ Among 75 singleton pregnancies with APS, women with multiple antibody positive reports were associated with worse outcomes including significantly lower live birth, and higher pregnancy-induced hypertension. Another retrospective study conducted using data obtained from the maternal-fetal clinic at Helen Schneider Hospital for women in Israel, also showed higher antibody titer in patients with APS to be associated with significantly higher premature birth.^[17]^ The association of several antibody titer with pregnancy outcomes in APS women has further been shown.^[18,19]^

Nevertheless, antithrombotic therapy including aspirin and heparin has shown to improve prognosis in these pregnant women with APS.^[20]^ Low dose aspirin along with heparin is preferred. However, heparin alone might also be associated with improved outcomes. In addition, hydroxychloriquine has also shown to improve pregnancy outcomes in such patients.^[21]^

### 4.1 Limitations

The limitations of this analysis have been stated: the number of participants with APS was limited, but at least sufficient to reach a fair conclusion. This autoimmune disorder is rare and therefore, it would require several years to attain a certain sufficient number of participants. Another limitation of this analysis might be the duration of disease period, and the treatment being given which were completely ignored in this research. As treatment and management change with time, treatment given in the year 1970 would definitely be different from treatment provided in 2015). This might influence the results and possibly affect the conclusion. Moreover, we have included 1 study with control group consisting of pregnant women with systemic lupus erythematous whereas in the other studies, the control group consisted of normal pregnant women. The study had to be included to increase the total number of suitable participants in the experimental group (the more the number of participants, the better the analysis, and hence, the better the conclusion). Few subgroups included only 2 studies’ postanalyses. This is because many studies reported outcomes which were different from each other. There were a few outcomes (including embolism, thrombocytopenia, amaurosis fugax, postpartum hemorrhage, myocardial infarction) which could not be assessed, since they were reported in only 1 study, lacking other study data for comparison. At last, another limitation might be the fact that the studies which were included in this analysis were prospective and retrospective ones, showing less effective data in comparison to randomized trials.

## 5 Conclusion

This analysis showed APS to be associated with significantly worse pregnancy outcomes when compared to the control group. A significantly higher risk of maternal and fetal complications was observed in this category of patients. Therefore, intense care should be given to pregnant women with APS to monitor unwanted outcomes and allow a successful pregnancy.

## Author contributions

LLP and SD were responsible for the conception and design, acquisition of data, analysis and interpretation of data, drafting the initial manuscript and revising it critically for important intellectual content. LLP wrote the final manuscript. All the authors approved the manuscript as written.

**Conceptualization:** Liping Liu, Dan Sun.

**Data curation:** Liping Liu, Dan Sun.

**Formal analysis:** Liping Liu, Dan Sun.

**Funding acquisition:** Liping Liu, Dan Sun.

**Investigation:** Liping Liu, Dan Sun.

**Methodology:** Liping Liu, Dan Sun.

**Project administration:** Liping Liu, Dan Sun.

**Resources:** Liping Liu, Dan Sun.

**Software:** Liping Liu, Dan Sun.

**Supervision:** Liping Liu, Dan Sun.

**Validation:** Liping Liu, Dan Sun.

**Visualization:** Liping Liu, Dan Sun.

**Writing – original draft:** Liping Liu, Dan Sun.

**Writing – review & editing:** Liping Liu, Dan Sun.
